# Nanofat lysate ameliorates pain and cartilage degradation of osteoarthritis through activation of TGF-β–Smad2/3 signaling of chondrocytes

**DOI:** 10.3389/fphar.2023.900205

**Published:** 2023-03-27

**Authors:** Yanzhi Ge, Wenting Xu, Zuxiang Chen, Haiyan Zhang, Wenbo Zhang, Junjie Chen, Jiefeng Huang, Wenxi Du, Peijian Tong, Letian Shan, Li Zhou

**Affiliations:** ^1^ The First Affiliated Hospital, Zhejiang Chinese Medical University, Hangzhou, Zhejiang, China; ^2^ Zhejiang Hospital, Hangzhou, Zhejiang, China; ^3^ Cell Resource Bank and Integrated Cell Preparation Center of Xiaoshan District, Hangzhou Regional Cell Preparation Center (Sanjiang Shangyu Biotechnology Co., Ltd.), Hangzhou, China; ^4^ Department of Rheumatism Immunology, Changhai Hospital, Navy Medical University, Shanghai, China

**Keywords:** osteoarthritis, Nanofat lysate, TGF-β–Smad2/3 signaling, chondrocytes, RNA sequencing

## Abstract

**Introduction:** Nanofat is an effective cell therapy for osteoarthritis (OA). However, it has clinical limitations due to its short half-life. We developed Nanofat lysate (NFL) to overcome the defect of Nanofat and explore its anti-OA efficacy and mechanism.

**Methods:** Monoiodoacetate (MIA) was employed to establish rat OA model. For pain assessment, paw withdrawal latency (PWL) and thermal withdrawal latency (TWL) were evaluated. Degeneration of cartilage was observed by histopathological and immunohistochemical examination. Primary chondrocytes were treated with TNF-α to establish the cellular model of OA. MTT, wound healing, and transwell assays were performed to assess effects of NFL on chondrocytes. RNA-seq, qPCR and Western blot assays were conducted to clarify the mechanism of NFL.

**Results and Discussion:** The animal data showed that PWL and TWL values, Mankin’s and OARSI scorings, and the Col2 expression in cartilage were significantly improved in the NFL-treated OA rats. The cellular data showed that NFL significantly improved the proliferation, wound healing, and migration of chondrocytes. The molecular data showed that NFL significantly restored the TNF-α-altered anabolic markers (Sox9, Col2 and ACAN) and catabolic markers (IL6 and Mmp13). The RNA-seq identified that TGF-β-Smad2/3 signaling pathway mediated the efficacy of NFL, which was verified by qPCR and Western blot that NFL significantly restored the abnormal expressions of TGFβR2, phosphorylated-Smad2, phosphorylated-Smad2/3, Col2, Mmp13 and Mmp3. After long-term storage, NFL exerted similar effects as its fresh type, indicating its advantage of storability. In sum, NFL was developed as a new therapeutic approach and its anti-OA efficacy and mechanism that mediated by TGF-β-Smad2/3 signaling was determined for the first time. Besides, the storability of NFL provided a substantial advantage than other living cell-based therapies.

## 1 Introduction

Osteoarthritis (OA) is the most common joint degenerative disorder and typically affects the knees, hands, and hips (Collins et al., 2019; Hunter and Bierma-Zeinstra, 2019; Xie et al., 2020). Its pathologic changes include destruction of the articular cartilage and inflammation, which eventually lead to progressive pain, loss of function, and generally reduced quality of life in OA patients (Krishnan and Grodzinsky, 2018). As a global disease, OA increases the burden of the whole society, and its indirect costs are estimated at 3.4–13.2 billion per year (Bateman et al., 2018). Multiple risk factors, such as aging, female sex, joint injury, and obesity, contribute to the occurrence and development of OA (Guermazi et al., 2017). Current treatments for OA mainly include pharmacological interventions (paracetamol, non-steroidal anti-inflammatory drugs, corticosteroid injections) and surgical approaches (joint replacement, osteotomy, joint distraction), with the aim of relieving pain and improving the motor ability of the affected joints (Wallace et al., 2019). However, these treatments bring unsatisfactory outcomes with serious adverse events in the clinic (Hunter and Bierma-Zeinstra, 2019). Therefore, exploring novel treatments for OA is critical.

Nanofat (also known as stromal vascular fraction) is obtained from the adipose tissue of the abdomen or inner thighs with enzymatic digestion. It contains multiple types of cells, such as the adipose stromal cells, adipose-derived stem cells, pericytes, endothelial progenitors, endothelial cells, and blood cells (Cross et al., 2014). Nanofat possesses pro-regenerative and anti-inflammatory activities, and its application has shown promising results in attenuating OA progression (Mehranfar et al., 2019; Perlman et al., 2019; Garza et al., 2020). In 16 OA patients, Nanofat significantly improved the functional outcome scores (VAS, WOMAC, and ROM) and promoted articular cartilage repair at 1-year follow-up visit (Hong et al., 2019). A systematic review of 200 OA patients reported that Nanofat relieved joint pain, improved functional ratings, and increased walking distance (Shanmugasundaram et al., 2021). Further studies have shown that the cells in Nanofat could interact with the microenvironment of OA, play an anti-inflammatory role on chondrocytes, and promote cartilage regeneration (Zhang et al., 2020). Owing to the autologous nature, availability, and little adverse effect of Nanofat, much attention has been devoted to its clinical application (Xu et al., 2019). However, Nanofat has to be used fresh, due to its unsuitability for storage and very short half-life period (Gentile et al., 2021; Rohani Ivari and Mahdipour, 2021). Therefore, the requirement for fresh preparations of autologous Nanofat brings repeated damage to patients, which has limited its clinical application and resulted in low patient compliance. In order to overcome this defect of Nanofat, we have developed Nanofat lysate (NFL) by repeated freeze–thawing lysis preparation on Nanofat. During the preparation, cell membranes of Nanofat cells are ruptured and their cellular contents released. Thus, NFL may have storage stability at a low temperature.

In this study, we developed Nanofat lysate (NFL) by repeated freeze–thawing lysis preparation on Nanofat for the first time. We evaluated the anti-OA efficacy of NFL by assessing joint pain, cartilage histology in monoiodoacetate (MIA)-induced OA rat model, and chondrocyte activity and inflammation response in a cellular model of OA. To explore the mechanism of NFL, RNA-seq (RNA sequencing) with the KEGG pathway analysis was applied, and qPCR and Western blot analyses were conducted for verification. Moreover, the influence of low-temperature storage on NFL’s anti-OA effects has been evaluated. This study would provide new knowledge on the anti-OA efficacy and mechanism of NFL and develop NFL as a superior cell therapy for OA treatment.

## 2 Materials and methods

### 2.1 Chemicals and reagents

Both Iscove’s Modified Dulbecco’s Medium (IMDM) and trypsin (0.25%) were purchased from Thermo Fisher Scientific (MA, United States). Fetal bovine serum (FBS) was purchased from CellMax (Beijing, China). Phosphate-buffered saline (PBS) was purchased from BasalMedia (Shanghai, China). MIA, 3-(4,5-dimethylthiazol-2-yl)-2,5-diphenyltetrazolium (MTT), and dimethyl sulfoxide (DMSO) were obtained from Sigma-Aldrich (Taufkirchen, Germany). Cell culture plates were purchased from Corning (NY, United States), and transwell chambers from Eppendorf (Hamburg, Germany). DNase I and TRIzol reagent were purchased from TaKaRa Biotechnology Co., Ltd. (Dalian, China). The cDNA Synthesis SuperMix kit was purchased from Biotool (TX, United States). The SYBR Green (2×) qPCR Master Mix (low ROX) kit was purchased from Bimake (TX, United States). Collagenase type I (0.2%) and collagenase type II (0.2%) were purchased from Worthington Biochemical Corp. (NJ, United States). All primary and secondary antibodies were obtained from Cell Signaling Technology Inc. (MA, United States).

### 2.2 Animal preparation

A total of 50 male Sprague-Dawley (SD) rats weighing 180–220 g were purchased from Shanghai Super B&K Laboratory Animal Co. Ltd. [Grade SPF II, SCXK (Shanghai): 2018-0006]. Therein, 10 rats were executed to acquire fat tissues from the lower abdomen, and the primary chondrocyte cells were isolated from the bilateral knee joints. The other 40 rats were housed under a controlled pathogen-free condition with 12 h day–night cycle, and fed dedicated fodder and filtered water before execution. The body weight of the rats was measured and recorded. All the experimental procedures were strictly in accordance with the Chinese legislation on the use and care of laboratory animals and approved by the Medical Norms and Ethics Committee of Zhejiang Chinese Medical University (Approval number: IACUC-20190506-14).

### 2.3 Nanofat and NFL preparation

Nanofat was obtained using a two-step centrifugation procedure consisting of collagenase I digestion, in accordance with the reported method (Tonnard et al., 2013). Briefly, after harvesting the fat tissue from the rats’ lower abdomen, the tissue was sliced into small pieces and digested with 0.2% collagenase I for 30 min at 37°C in the ThermoMixer (Eppendorf). After centrifugation at 210 × *g* for 5 min, cell pellets were obtained and resuspended in PBS, followed by filtration through a cell strainer (100 μm). The collected solution was washed using PBS thrice and concentrated to obtain Nanofat concentrates. The cell number in Nanofat was measured by a blood cell analyzer (Mindray BC-3000 plus, Shenzhen, China) and standardized to 1 × 10^6^ cells/mL. Acquired Nanofat cells were treated with freeze–thawing (−80°C to 37°C) cycles thrice, followed by centrifugation at 2,000 × *g* for 5 min to remove residual fragments. The obtained supernatant was designated as NFL and stored at −80°C before use. Sketch map of NFL production was shown in Supplementary Figure S4.

### 2.4 OA modeling and intervention

The 40 SD rats were randomly divided into four equal groups: normal control (NC) group, model group, Nanofat group, and NFL group. For the NC group, 10 rats were intra-articularly injected with 50 μL of saline in knee joints. The rats in the remaining three groups were intra-articularly injected with 50 μL MIA (30 mg/mL) per knee joint for the establishment of the OA model (Lee et al., 2018). After OA modeling for 7 days, the rats in the Nanofat group and NFL group were intra-articularly injected with 10^5^ Nanofat cells and 10^5^ Nanofat-derived NFL resuspended in 50 μL PBS, respectively, by a weekly injection for four times in all. Meanwhile, the rats in the NC group were intra-articularly injected with 50 μL PBS in both knees. Inflammation development was evaluated by measuring knee swelling. The knee diameter changes on different days were indicated by using a Vernier caliper.

### 2.5 Pain behavior assessment

The pain behavior parameters (PWL and TWL) were measured by a plantar test apparatus (Ugo Basile, Varese, Italy) with the von Frey method (Chaplan et al., 1994; Yan et al., 2018). Specifically, daily in the morning, each rat was separately placed in a 10^3^ cm individual plastic chamber for 30 min acclimatization, followed by a needling stimulation at the plantar surface of the hind paws, thrice at an interval of 5 min. The number of pressure (g) shown in the screen was recorded as PWL. For TWL measurement, a precision-focused beam of radiant heat (60°C) was irradiated to the same paw district and the withdrawal latency of the escaped paw(s) was measured by machine. TWL was tested thrice at 5 min intervals and recorded by a new examiner blinded to the groups.

### 2.6 Blood routine analysis

Blood samples were collected from the heart of the rats after anesthesia, and the blood cell analyzer was used to detect blood cell indexes (Shenzhen Mindray Medical Devices Co. Ltd.), such as those of white blood cells, monocytes, lymphocytes, and neutrophils.

### 2.7 Histopathological and immunohistochemical analysis

All rats were sacrificed after the pain behavior experiment. The knee joints from each of the rats were acquired and fixed with 5% buffered paraformaldehyde for 72 h, followed by decalcifying with 14% EDTA, lasting for a month. Each sample was then embedded in paraffin, sectioned (3 µm), and collected on microscopic slides. After staining with hematoxylin and eosin (HE) and Safranin-O (SO), the images of staining were pictured by a light microscope (Axio Scope A1, ZEISS, Germany) and analyzed with the Image-Pro Plus (IPP) 6.0 software (Media Cybernetics, MD, United States). Pathologic findings of the OA progression were evaluated by Mankin’s and OARSI scoring systems by double-blind observations (Mankin et al., 1971; Glasson et al., 2010). Anti-Col2 (Novusbio, NB600-844, 1:500) antibodies were performed on a 3-μm-thick tissue section, and the immunoreactivity was semi-quantified by using the IPP software.

### 2.8 Primary chondrocytes isolation

After the extraction of Nanofat as previously stated, both knees and hips of the rats were dissected to acquire articular cartilage tissues (Ma et al., 2021). The lucency tissues were carefully excised, washed thrice using PBS containing 100 U/mL penicillin, and placed into a new culture dish. The harvested tissues were sliced into 1 mm³ small pieces by an ophthalmic scissor and digested with 0.25% trypsin for 30 min and 0.5% collagenase II for 4 h, in a 37°C and 5% CO_2_ incubator. A 70 μm cell strainer was used after digestion to filter the isolated chondrocytes. All the operations mentioned above were carried out under sterile conditions on a super clean bench. IMDM containing 10% FBS and 100 U/mL penicillin was used to culture the cells. The cells were split into the third passage before use for the following experiments. The cultured chondrocytes from the same passage were randomly divided into three groups with different interventions as follows: NC group (treated with saline), TNF-α group (treated with 10 ng/mL TNF-α for 6 h), and TNF-α+NFL group (treated with 10 ng/mL TNF-α for 6 h followed by NFL derived from 1 × 10^5^ Nanofat for another 24 h). To explore whether the storage condition affects the efficacy of the NFL, the TNF-α+NFL group was further separated into three subgroups after −80°C preservation at three different time points (0, 8, and 12 weeks).

### 2.9 Cell viability assay

The MTT assay was used to detect the cell viability of the cultured chondrocytes. After the initial treatment of TNF-α for 24 h, rat chondrocytes were seeded in a 96-well plate (1 × 10^4^ chondrocytes/well). Anchorage-dependent cells were treated with NFL (1 × 10^3^, 4 × 10^3^, 1.6 × 10^4^, 6.4 × 10^4^, and 2.56 × 10^5^) for another 24 h and 48 h. 20 μL MTT working solution was added to each well, and the plates were further incubated at 37°C for 4 h. The optical density (OD) values were measured at 490 nm using a Bio-Rad microplate reader (Hercules, CA, United States). The proliferative rate (%) = [(treated OD − untreated OD)/untreated OD] × 100.

The cell viability of chondrocytes was determined by the CCK-8 assay at 24 and 48 h in the Supplementary Figure S2. Briefly, the chondrocytes were seeded in 96-well plates at a density of 5 × 10^3^ cells/well in 200 μL medium for 24 h, The cultured chondrocytes from the same passage were randomly divided into five groups with different interventions as follows: NC group (treated with saline), TNF-α group (treated with 10 ng/mL TNF-α for 6 h), TNF-α+NFL group, TNF-α+Exo group and TNF-α+TP group (treated with 10 ng/mL TNF-α for 6 h followed by NFL, exosome or total protein from 10^5^ Nanofat respectively, for another 24 or 48 h). Aliquots of each 20 μL CCK-8 solution were added to each well and incubated at 37°C for 2 h, until the color turned to orange. The OD value was measured at 450 nm with a microplate reader. The proliferative rate was calculated in the same way as MTT assay.

### 2.10 Wound healing assay

To perform the wound healing assay, the cultured cells were equally seeded into a 6-well plate (3 × 10^5^ chondrocytes/well). Each well was scratched in the central part in a cross form after the confluence reached 90%, followed by treatment of NFL in FBS-free IMDM. The cells were observed and imaged at four different time points (0, 24, 48, and 72 h) under an inverted microscope (Carl Zeiss, Göttingen, Germany). The wound area was calculated with the ImageJ (version: 1.8.0) software. Each experiment was conducted in triplicate.

### 2.11 Transwell migration assay

A 24-well transwell dish with 8 μm nitrocellulose pore filters was used to perform a transwell migration assay. Aliquots of 200 μL rat chondrocytes (2 × 10^5^ cells/mL) in IMDM were loaded into the upper chamber, while 600 μL α-MEM medium with Nanofat and NFL into the lower chamber for the Nanofat group and NFL group, respectively. The NC group applied only α-MEM in the lower chamber. All media used in the upper and lower chambers were serum free. After incubation for 18 h, the chondrocytes those passed through the membranes were fixed with 4% paraformaldehyde (Servicebio, Wuhan, China) and stained with 1% crystal violet dye solution (Beyotime Biotechnology, Shanghai, China). The images of each group were captured by a microscope in different areas in three independent repeated experiments.

### 2.12 qPCR analysis

The mRNA expression of the targeted genes in the chondrocytes was measured using a real-time PCR (qPCR) assay on the QuantStudio™ 7 Flex Real-Time PCR system (Thermo Fisher Scientific, United States). Total RNA was extracted with TRIzol and the quality was controlled by the NanoDrop 2000 spectrophotometer (Thermo Fisher Scientific, United States). Genomic DNA was removed using the DNase I kit. cDNA reverse transcription was performed by using the All-in-One cDNA Synthesis SuperMix. The PCR reaction system was 20 μL, containing 10 μL 2× SYBR (Tli RNaseH Plus), 8.2 μL ddH_2_O, 1 μL template cDNA, 0.4 μL forward primer, 0.4 μL reverse primer, with the following reaction conditions: initial denaturation at 95°C for 5 min, 40 cycles of denaturation at 95°C for 3 s, and annealing and extension at 60°C for 30 s. *β-Actin* was used as the reference gene, and the 2^−ΔΔCT^ method was used to analyze the relative mRNA expressions. A list of primer sequences of the target genes is shown in Table 1.

**TABLE 1:** Primer sequences used for qPCR analysis.

Genes	Forward primer	Reverse primer
*β-Actin*	5′-CCC​GCG​AGT​ACA​ACC​TTC​T-3′	5′-CGT​CAT​CCA​TGG​CGA​ACT-3′
*Sox9*	5′-TCC​AGC​AAG​AAC​AAG​CCA​CA-3′	5′-CGA​AGG​GTC​TCT​TCT​CGC​TC-3′
*ACAN*	5′-GCA​GAC​ATT​GAT​GAG​TGC​CTC-3′	5′-CTC​ACA​CAG​GTC​CCC​TCT​GT-3′
*Col2*	5′-CTC​AAG​TCG​CTG​AAC​AAC​CA-3′	5′-GTC​TCC​GCT​CTT​CCA​CTC​TG-3′
*IL-1β*	5′-TGT​GGC​AGC​TAC​CTA​TGT​CT-3′	5′-GGG​AAC​ATC​ACA​CAC​TAG​CA-3′
*IL6*	5′-CCT​CTG​GTC​TTC​TGG​AGT​ACC-3′	5′-ACT​CCT​TCT​GTG​ACT​CCA​GC-3′
*Mmp1*	5′-CCA​GGT​ATT​GGA​GGG​GAT​GC-3′	5′-CCA​AGG​GAA​TGG​CCC​AGT​T-3′
*Mmp13*	5′-CTA​TGG​TCC​AGG​AGA​TGA​AGA​C-3′	5′-GTG​CAG​ACG​CCA​GAA​GAA​TCT-3′
*Adamts-5*	5′-TGG​AGT​GTG​TGG​AGG​GGA​TA-3′	5′-CGG​ACT​TTT​ATG​TGG​GTT​GC-3′
*TGF*β*R2*	5′-GTG​TTT​TCA​TCA​TGC​TGG​CT-3′	5′-CGT​GAA​GTG​GCT​GTT​GAT​CT-3′

### 2.13 RNA sequencing analysis

The extraction, quality control, and concentration of total RNA from the three groups of chondrocytes were performed as stated before (Ma et al., 2019). Each sample was prepared with three replicates. RNA purification, reverse transcription, library construction, and RNA-seq detection were conducted by BGI Corporation (Shenzhen, China) according to the manufacturer’s instructions (MGISEQ-2000). To identify the differentially expressed genes (DEGs) between two different samples, the expression level of each transcript was calculated by the fragments per kilobase of exon per million mapped reads method. The functional enrichment analysis was based on the Kyoto Encyclopedia of Genes and Genomes (KEGG) database, and the enriched DEGs at *p*-value < 0.05 among the three groups (NC *vs*. TNF-α and TNF-α *vs*. TNF-α+NFL) were used for the pathway analysis.

### 2.14 Western blot analysis

Total protein of the chondrocytes in 10 cm plates was extracted with lysis buffer (50 mM Tris-HCl, pH 7.4, 150 mM NaCl, 1 mM EDTA, 1% Triton, and 0.1% SDS) containing proteinase inhibitor cocktail (Bimake, Houston, TX, United States) on ice for half an hour. The targeted protein was separated with 10% denaturing sodium dodecyl sulfate–polyacrylamide gel electrophoresis and transferred onto a nitrocellulose membrane (Corning Costar, NY, United States). After blocking with 5% BSA at 4°C for 2 h, the membrane was incubated with the targeted primary antibodies (anti-β-actin, anti-Mmp3, anti-Mmp13, anti-Col2, anti-Smad2, anti-phospho-Smad2, anti-Smad2/3, anti-phospho-Smad2/3, and anti-TGFβR2, 1:1,000 dilution) overnight at 4°C. After washing with TBST thrice, the membranes were incubated with horseradish peroxidase (HRP)–conjugated anti-mouse/anti-rabbit secondary antibodies at 4°C for 2 h. All protein bands were detected by the ECL kit (Biosharp, Beijing, China) and visualized by the GE ImageQuant LAS 4000 System (Bio-Rad, Hercules, CA, United States). β-Actin was used as the internal normalization control. The results were analyzed with the ImageJ software (version: 1.8.0).

### 2.15 Statistical analysis

Data are presented as mean ± standard deviation (SD). The data from different groups were intercompared using one-way ANOVA followed by the Fisher’s least significant difference (LSD) method, and the SPSS (version: 22.0) software was used to process the data. * and ^#^ indicate a significant difference of *p* < 0.05, ** and ^##^ indicate a very significant difference of *p* < 0.01, and *** indicates an extremely significant difference of *p* < 0.001.

## 3 Results

### 3.1 Anti-OA efficacy of NFL in rats

**FIGURE 1:**
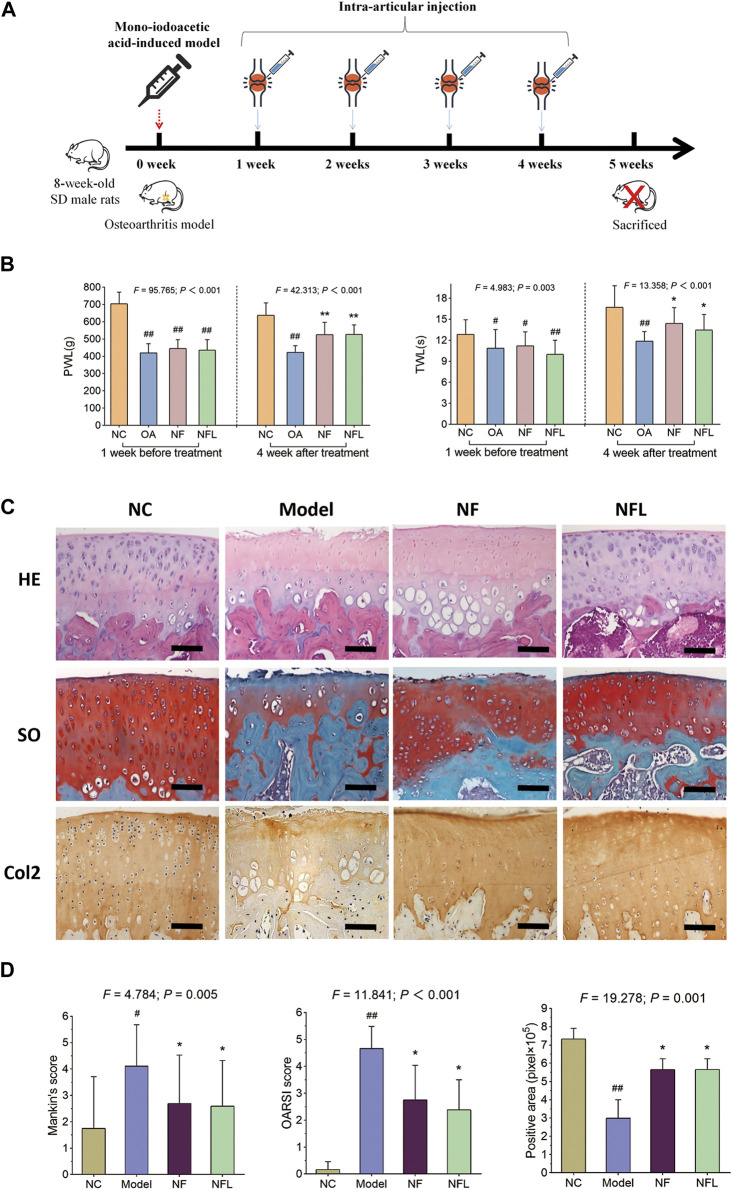
Study design of the animal experiment **(A)**; pain behavior assessment (TWL and PWL) before and after treatment **(B)**; histopathological observation on cartilage by HE and SO staining and immunohistochemical observation of Col2 expression **(C)**; Quantification of Mankin’s and OARSI scores, and positive area of Col2 expression **(D)**. Values are shown as mean ± SD. ^#^ *p* < 0.05 or ^##^ *p* < 0.01 *vs*. NC group; ^*^ *p* < 0.05 or ^**^ *p* < 0.01 *vs*. Model group. NF, Nanofat. Scale bar = 100 μm.

To evaluate the *in vivo* efficacy of NFL, an OA rat model was established and treated with intra-articular injection of NFL (Figure 1A). As shown in Figure 1B, PWL and TWL are significantly decreased with OA modeling (each *p* < 0.05 *vs*. NC) and significantly increased by NFL and Nanofat (each *p* < 0.05 *vs*. Model). As shown in Figure 1C, a rough cartilage surface, hypertrophy and apoptosis of the chondrocytes, and a decrease of cartilage thickness are observed in the OA rat joints, while the surface smoothness of the cartilage, number and phenotype of the chondrocytes, and the cartilage thickness are significantly improved by NFL and Nanofat treatments. As shown in Figure 1D, the Mankin′s and OARSI scores demonstrate that NFL and Nanofat have significantly reversed the OA modeling–induced abnormal histopathological scores (each *p* < 0.05 *vs*. Model). In addition, Col2 immunohistochemical staining shows that the Col2 expression (positive area) are significantly decreased in the model (*p* < 0.05 *vs*. NC) but are significantly reversed by the treatments (each *p* < 0.05 *vs*. Model). The above effects of NFL and Nanofat do not differ from each other. The results indicate that intra-articular injections of NFL and Nanofat exerted comparable and substantial anti-OA efficacy *in vivo*.

### 3.2 Proliferative effect of NFL on chondrocytes

**FIGURE 2:**
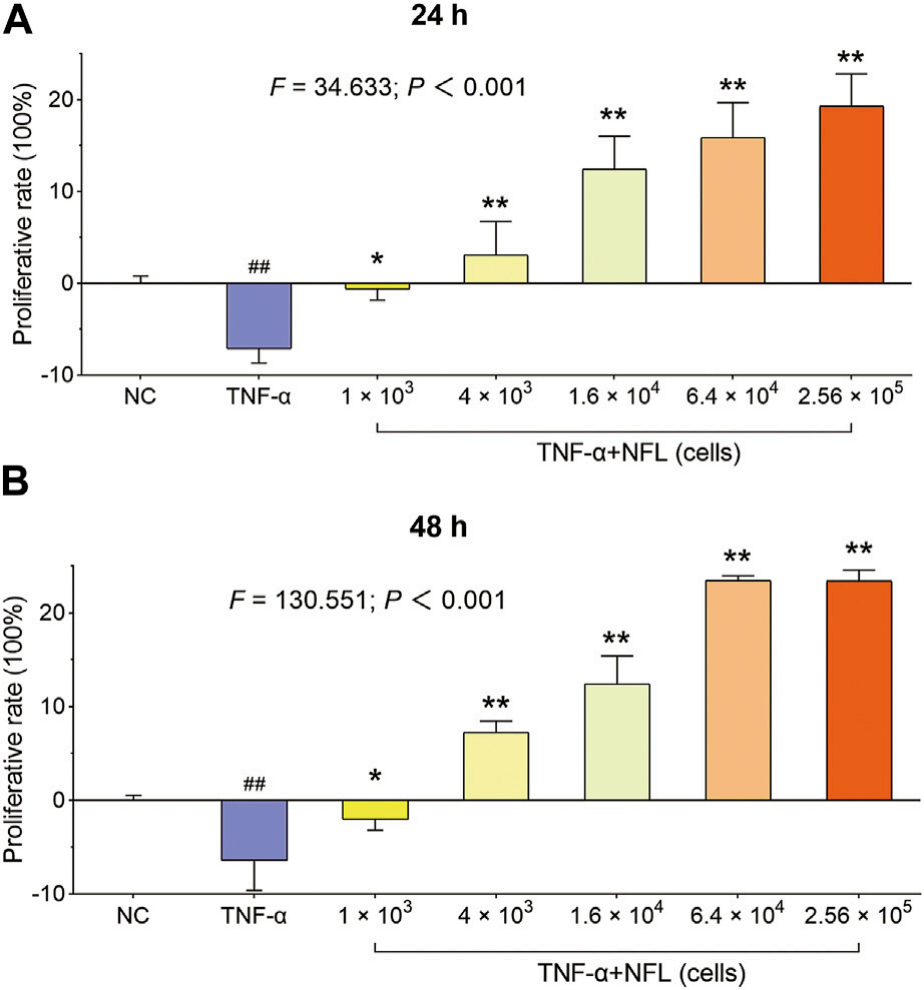
The cell viability of chondrocytes after NFL treatment. **(A)** Proliferative rate of chondrocytes at 24 h; **(B)** Proliferative rate of chondrocytes at 48 h. Values are shown as mean ± SD. ^##^ *p* < 0.01 *vs*. NC group; *p < 0.05 or **p < 0.01 *vs*. TNF-α group. Scale bar = 200 μm.

Unlike Nanofat, which has a cellular morphology, NFL is a clear and transparent fluid. The isolated chondrocytes were polygonal, with abundant and uniform cytoplasm, large and round nuclei, and interconnected with each other in a cobblestone-like structure. The proliferative effect of NFL on the chondrocytes was evaluated by the MTT assay. As shown in Figure 2, the cell viability of chondrocytes is significantly decreased with TNF-α treatment at 24 and 48 h (*p* < 0.01 *vs*. NC), while the decrease is reversed by NFL derived from 1 × 10^3^ to 2.56 × 10^5^ Nanofat cells. The proliferative rate of the chondrocytes is significantly increased from −7.12% ± 1.57% to 19.28% ± 3.53% after the 24-h treatment (Figure 2A, *p* < 0.05 or *p* < 0.01 *vs*. TNF-α group) and from −6.45% ± 3.18% to 23.38% ± 1.17% after the 48-h treatment (Figure 2B, *p* < 0.05 or *p* < 0.01 *vs*. TNF-α group) in a dose-dependent manner.

### 3.3 Effects of NFL on wound healing and migration of chondrocytes

**FIGURE 3:**
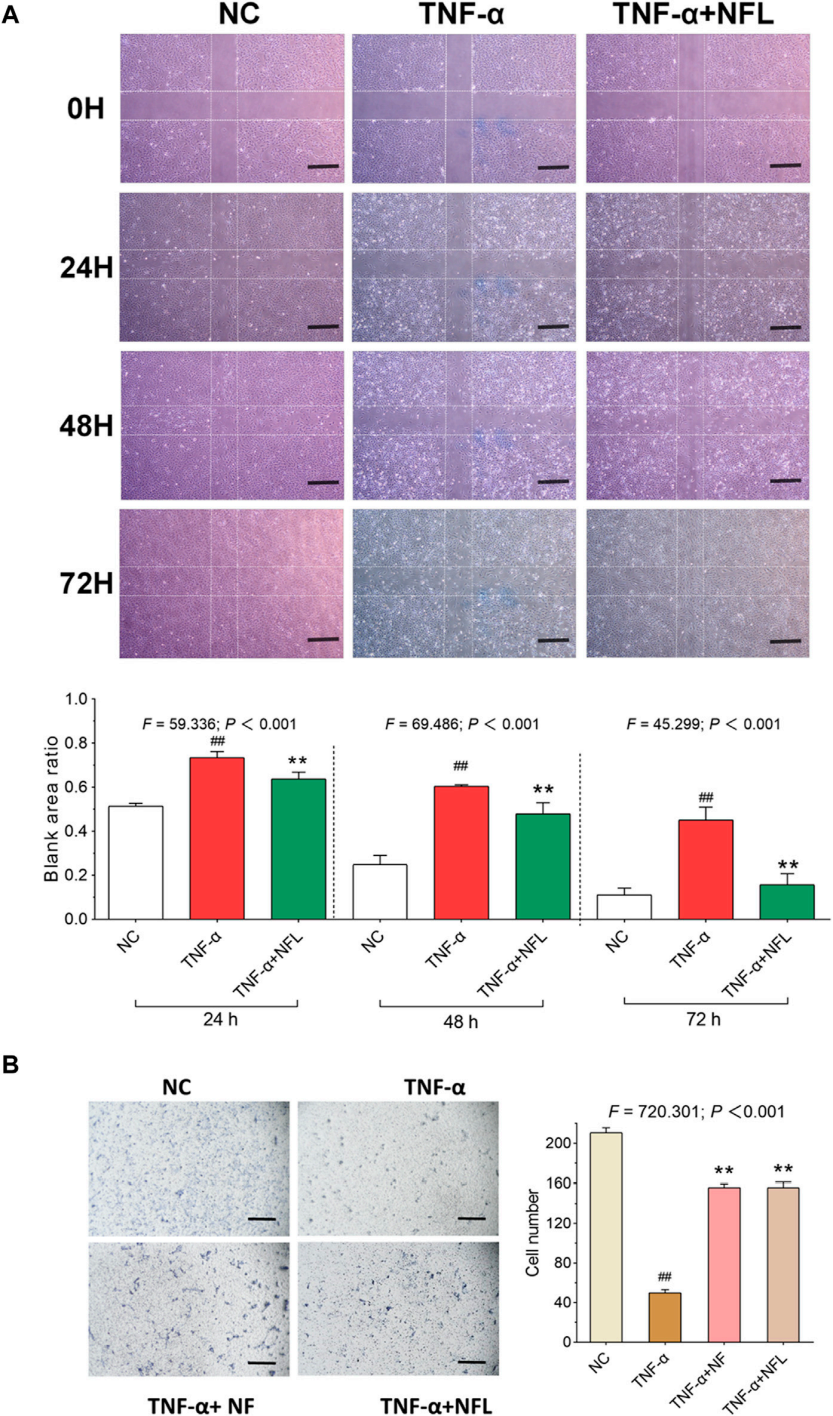
Wound healing and cell migration assays on chondrocytes with NFL treatment. **(A)** Wounding healing assay of chondrocytes with NFL treatment at 24, 48, and 72 h, and wound healing rate represented as the ratio of the scratched wound area at 24, 48, and 72 h treatment to the area without treatment (0 h). **(B)** Transwell assay of chondrocytes with NF and NFL intervention. Values are shown as mean ± SD. ^##^ *p* < 0.01 *vs*. NC group; **p < 0.01 *vs*. TNF-α group. NF, Nanofat. Scale bar = 200 μm.

Wound healing and transwell assays were conducted to evaluate the migration of chondrocytes with NFL treatment. As shown in Figure 3A, the blank area ratio is significantly increased after adding TNF-α (*p* < 0.01 *vs*. NC) and significantly decreased after NFL treatment at 24, 48, and 72 h (each *p* < 0.01 *vs*. TNF-α group). As shown in Figure 3B, the migrated number of chondrocytes to the bottom of chamber is significantly decreased after adding TNF-α (*p* < 0.01 *vs*. NC) and significantly increased after NFL treatment (each *p* < 0.01 *vs*. TNF-α group). Moreover, no significant difference is found between NFL and Nanofat in the result of the transwell assay.

### 3.4 Molecular actions of NFL on mRNA expressions of chondrocytes

**FIGURE 4:**
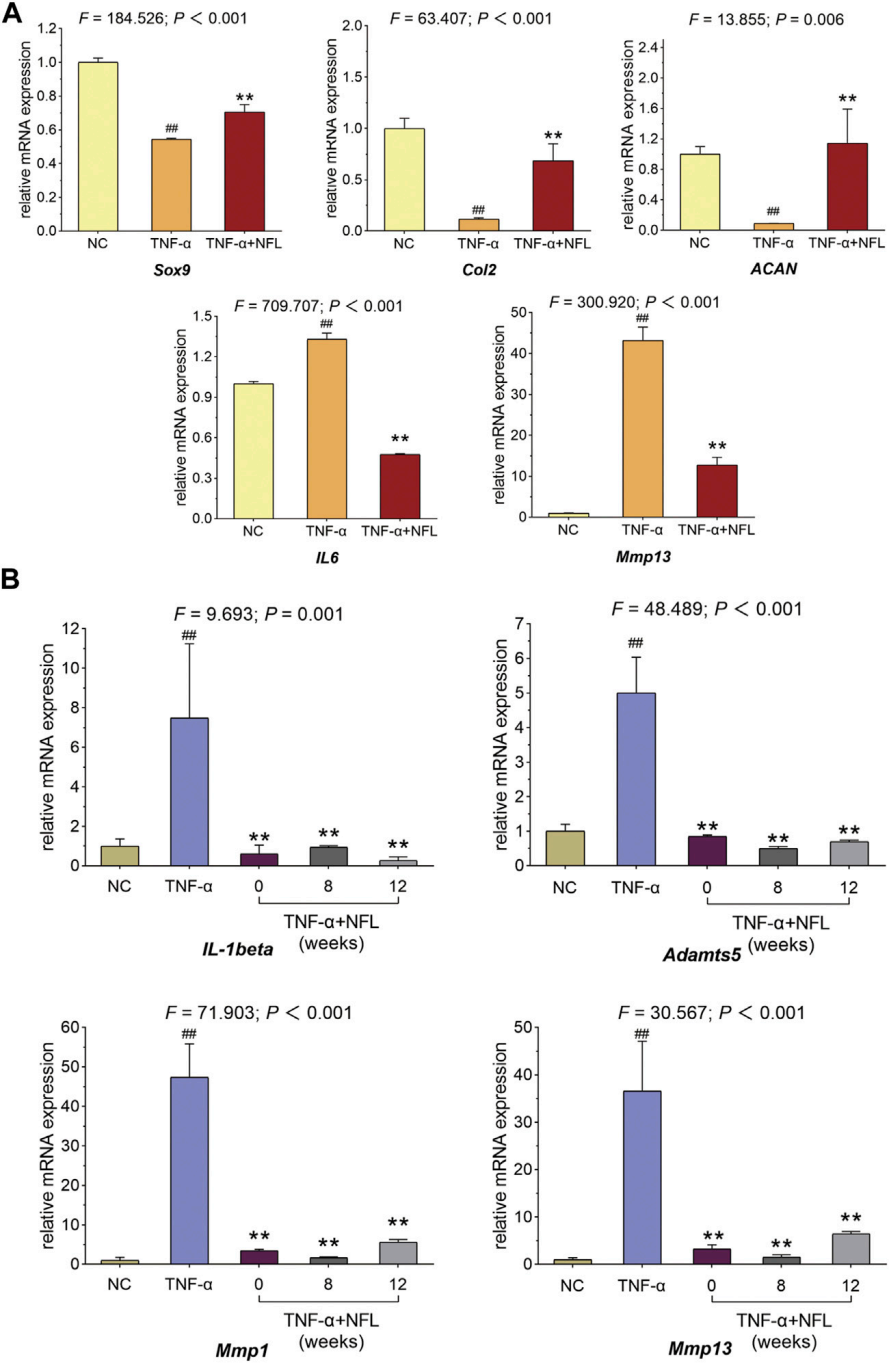
qPCR assay on chondrocytes with NFL and sNFL treatments. **(A)** Relative mRNA expressions of selected genes in chondrocytes with NFL treatment. **(B)** Relative mRNA expressions of selected genes in chondrocytes with NFL and sNFL treatments. Values are shown as mean ± SD. ^##^ *p* < 0.01 *vs*. NC group; **p < 0.01 *vs*. TNF-α group.

The qPCR assay was applied to evaluate the relative mRNA expressions of the genes related to OA pathology and inflammation in chondrocytes. As shown in Figure 4A, the mRNA expressions of *Sox9*, *Col2*, and *ACAN* are significantly downregulated while those of *IL6* and *Mmp13* are significantly upregulated by TNF-α in the model group (each *p* < 0.01 *vs*. NC). These pathological alterations are significantly reversed by NFL after the 48-h treatment (each *p* < 0.01 *vs*. TNF-α group). To evaluate the storage influence on NFL, inflammation-related genes *Mmp13*, *Mmp1*, *Adamts-5*, and *IL-1β* have been assessed after stored NFL (sNFL) treatment at three storage time points (0, 8 and 12 weeks). As shown in Figure 4B, sNFL stored for 12 weeks has significantly decreased the mRNA expression of *Mmp13*, *Mmp1*, *Adamts-5*, and *IL-1β* (each *p* < 0.01 *vs*. TNF-α group) as fresh NFL without storage.

### 3.5 RNA-seq with KEGG pathway analysis

**FIGURE 5:**
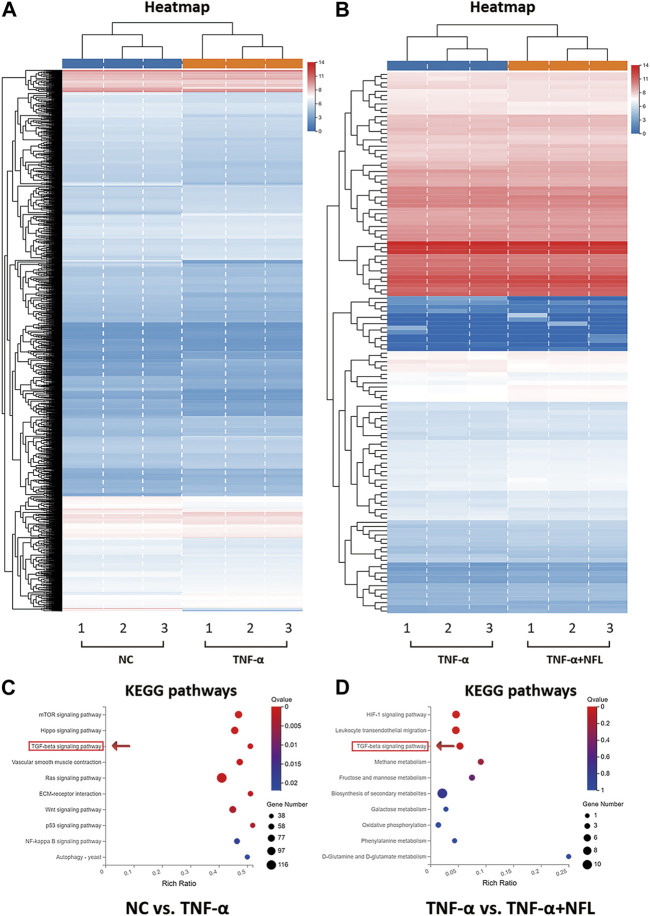
RNA-seq analysis of chondrocytes treated by NFL. **(A)** Heatmap of DEGs between NC and TNF-α groups; **(B)** Heatmap of DEGs between TNF-α and TNF-α+NFL groups. Red rectangles represent upregulation, and blue rectangles represent downregulation. Each column of the same group indicates an independent biological replicate (*n* = 3); **(C)** KEGG analysis of DEGs between NC and TNF-α groups; **(D)** KEGG analysis of DEGs between TNF-α and TNF-α+NFL groups. The color key from blue to red represents the *q* value from high to low and the size of the bubble indicates the gene number.

The RNA-seq method based on transcriptome analysis was applied to investigate the molecular alterations in chondrocytes among the NC, TNF-α, and TNF-α+NFL groups. The statistical analysis revealed a total of 2,833 upregulated genes and 2,994 downregulated genes between the NC and TNF-α groups (*p* < 0.05, Figure 5A), and 49 upregulated genes and 79 downregulated genes between the TNF-α and TNF-α+NFL groups (*p* < 0.05, Figure 5B). The details of DEGs are shown in supplementary Table 1. The significantly upregulated and downregulated genes were functionally analyzed using the KEGG database. The significantly relevant pathways annotated by the KEGG pathway analysis are shown in Figures 5C, D. Of these, the TGF-β signaling pathway was inhibited by TNF-α and activated by NFL, which was screened out as the mediator for NFL’s mechanism.

### 3.6 Verification of TGF-β–Smad2/3 signaling–mediated mechanism of NFL

**FIGURE 6:**
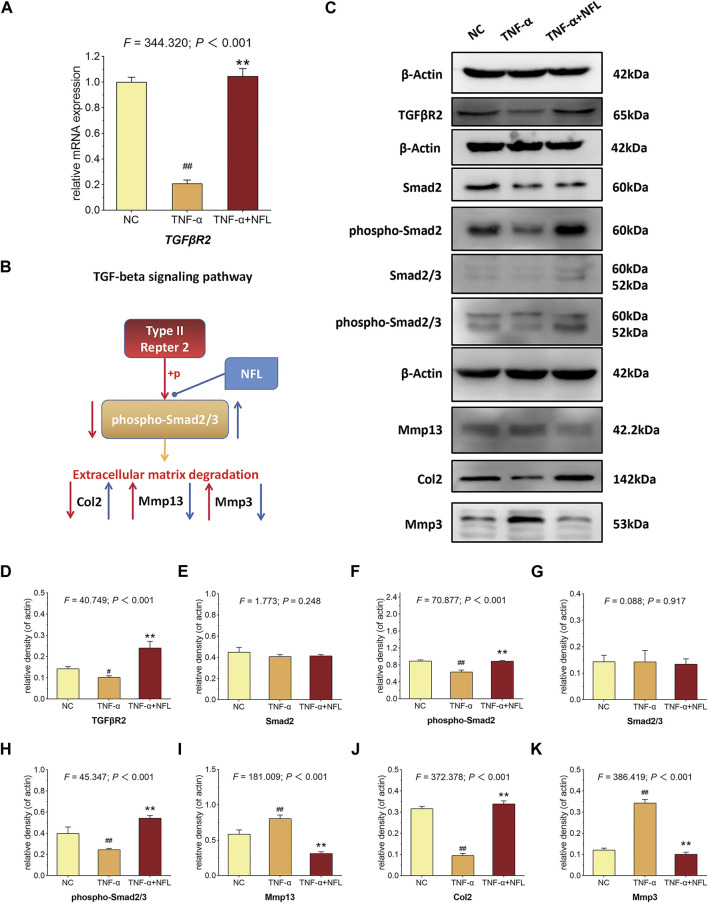
qPCR verification and Western blot analysis of chondrocytes in TGF-β–Smad2/3 signaling pathway. **(A)** The relative mRNA expression of *TGFβR2* was tested to verify the RNA-seq result; **(B)** deduction of TGF-β–Smad2/3 signaling–mediated mechanism; **(C)** Western blot assay of indicated proteins and the statistical analysis of relative density for **(D)** TGFβR2, **(E)** Smad2, **(F)** phospho-Smad2, **(G)** Smad2/3, **(H)** phospho-Smad2/3, **(I)** Mmp13, **(J)** Col2 and **(K)** Mmp3. ^#^ *p* < 0.05 or ^##^ *p* < 0.01 *vs*. NC group; **p < 0.01 *vs*. TNF-α group.

According to the RNA-seq result, the TGF-β signaling pathway was picked for verification. As shown in Figure 6A, TNF-α has significantly decreased the mRNA expression of *TGFβR2* (*p* < 0.01 *vs*. NC), while NFL has significantly reversed the altered expression (*p* < 0.01 *vs*. TNF-α group). As shown in Figures 6C–K, TNF-α has significantly downregulated the protein expression of TGFβR2, phospho-Smad2, phospho-Smad2/3, and Col2 (*p* < 0.05 of *p* < 0.01 *vs*. NC), while NFL has significantly upregulated the protein expression of Mmp13 and Mmp3 (*p* < 0.01 *vs*. NC). The results have confirmed that the TGF-β–Smad2/3 signaling pathway mediates the effects of NFL on the chondrocytes as we predicted (Figure 6B).

## 4 Discussion

Nanofat is always used in an autologous manner, which avoids the ethical concerns about the *ex vivo* cell expansion and can be directly applied at the bedside. Thus, Nanofat is more feasible and applicable for clinical application than living cell–based therapies (e.g., stem cell therapy). Recently, clinical studies have demonstrated the therapeutic value of Nanofat in OA treatment, and a single intra-articular injection of Nanofat could significantly improve joint pain and function of OA patients and provide an alternative to surgical interventions (Vinet-Jones and Darr, 2020; Bolia et al., 2021). The adipose-derived stem cells, endothelial progenitor cells, macrophages, fibroblasts, pericytes, and stromal cells are cell components contained by Nanofat, which excrete various cytokines, chemokines, and growth factors to play paracrine roles in tissue regeneration (Dicker et al., 2005). Epithelial–mesenchymal transition (EMT) is a process in which epithelial cells transform into mesenchymal cells, which is involved in embryogenesis, wound healing, and malignant progression of tumors. The role of macrophages in inducing EMT *via* secretion of a distinct cohort of cytokines and chemokines has been extensively studied. Tumor-associated macrophages (TAMs) secrete TGFβ, which acts in a paracrine fashion, much like the TGFβ secreted by CAFs, to induce EMT in breast, hepatocellular, and F9 teratocarcinoma cells. In addition, within lung cancer cells, IL-6 secreted by TAMs activates the cyclooxygenase 2 (COX2), prostaglandin E2 (PGE2), and β-catenin signaling pathways, which activate EMT and cell invasion. However, the cells in Nanofat have a short half-life period and cannot be easily stored for a long time (Xu et al., 2019). Therefore, the autologous application of Nanofat is clinically limited, since Nanofat has to be used fresh and the repeated injections in one course of Nanofat treatment means repeated damage to patients by adipose sampling. To address this issue, we developed NFL on the basis of the paracrine action of Nanofat. After freeze–thawing lysis, the excreted cytokines, chemokines, and growth factors as well as intracellular bioactive substances of Nanofat cells were extracted and collected, which could be easily stored in a low temperature refrigerator or liquid nitrogen. Moreover, the quality control of NFL, which doesn’t contain any living cells, would be more convenient and efficient. In this study, we developed Nanofat lysate (NFL) by repeated freeze–thawing lysis preparation on Nanofat for the first time. We compared the anti-OA efficacy between NFL and Nanofat and demonstrated that NFL exerted similar effects to Nanofat on joint pain and cartilage degradation (Figure 1), without obvious side effects on animals (Supplementary Figures S1A, B). Furthermore, we discovered that NFL exerted chondroprotective effects of improving cell viability, wound healing, cell migration, and anabolism/catabolism balance of chondrocytes to resist inflammatory stress (Figures 2–4). TGF-β inhibited chondrocyte hypertrophy and antagonized the activity of inflammatory factors by activating ALK5 to mediate the phosphorylation of Smad2/3, which was stimulated by NFL in the chondrocytes (Supplementary Figures S3A, B). The underlying mechanism of NFL was then clarified to be due to the reactivation of the TGF-β–Smad2/3 signaling pathway (Figures 5, 6). NFL composition is complicated and contains exosomes, proteins and some other substances. We tried to figure out the effective components of NFL. The NFL was centrifuged at ultra-high speed to separate the exosomes (Exo) from the rest of the fraction, which was so called total protein (TP) in this study. Results from CCK-8 and Western blot analysis showed that Exo and TP were as effective as NFL in stimulating cell proliferation and TGF-β-Smad2/3 signaling (Supplementary Figures S2, S3), indicating that components in NFL may have synergistic chondro-protective effect. When we stored NFL at −80°C for 12 weeks, the effects of the stored NFL on anabolism/catabolism balance of chondrocytes did not significantly differ from that of fresh NFL (Figure 4), indicating to the long-term storability of NFL and a possibility for repeated use with only one sampling. When compared with the NC group, the whole-blood lymphocyte count increased after intra-articular Nanofat injection, indicating inflammation or infection, while NFL injection did not increase the lymphocyte count (Supplementary Figure S1). NFL and Nanofat were homologous biomaterials derived from the adipose tissue. Our previous study has demonstrated the anti-OA efficacy of Nanofat and its paracrine mode of action (Chen et al., 2021).

The TGF-β/Smad signaling pathway has been reported to play critical roles in OA occurrence and development (Shen et al., 2014). This pathway indispensably participates in all stages of chondrocyte proliferation, chondrogenesis, and extracellular matrix deposition (Tuli et al., 2003; Song et al., 2007). Our RNA-seq and molecular experimental data have confirmed that the TGF-β/Smad signaling pathway was downregulated during the process of OA and upregulated with NFL treatment, in which Smad2/3 played the key role. The finding is to some extent in line with previous reports on the role played by the TGF-β signaling pathway in the process of OA (Roman-Blas et al., 2007). Smad2/3 are the core inhibitory mediators of the TGF-β pathway participating in the process of chondrocyte terminal differentiation, and the differentiated chondrocytes eventually become hypertrophic with disruption of anabolic/catabolic balance, e.g., overexpression of Mmp13 and low expression of Col2 (van der Kraan et al., 2009). We have revealed that NFL significantly increased the low expressions of anabolic molecules (Col2, Aggrecan, Sox9) and suppressed the overexpression of catabolic molecules (Mmp13, Mmp3, Mmp1, Adamts-5, IL-1β, and IL-6) with the activation of TGFβR2 and phosphorylation of Smad2/3, indicating that NFL attenuated the inflammatory progression of OA by reactivating the TGF-β signaling pathway through phosphorylation of Smad2/3.

The main innovation points of this study include 1) the development of NFL by freeze–thawing lysis of Nanofat for OA treatment; 2) the determination of a similar anti-OA efficacy between NFL and Nanofat; 3) the determination of the long-term storability of NFL without the loss of therapeutic potential on OA; and 4) the clarification of TGF-β–Smad2/3 signaling pathway–mediated mechanism of NFL. Nevertheless, there are several limitations to this study: 1) the therapeutic material basis (e.g., cytokines, chemokines, and growth factors) of NFL was unrevealed; 2) the evaluation of long-term stored NFL was only regarding anabolic/catabolic balance at the molecular level, which is not sufficient; and 3) the RNA-seq data showed many relevant signaling pathways but only the TGF-β signaling pathway was verified. Further studies are required to address the above issues and complement the knowledge of NFL for its clinical application.

## 5 Conclusion

This study developed NFL from Nanofat and demonstrated its therapeutic efficacy of relieving joint pain and cartilage degradation against OA in rats as well as chondroprotective activity of improving cell viability, wound healing, cell migration, and the anabolism/catabolism balance against inflammatory stress in chondrocytes. The TGF-β–Smad2/3 signaling pathway was found to mediate such a mechanism of NFL. The anti-OA efficacy of NFL was similar to that of Nanofat, and the stored NFL at −80°C for 12 weeks still exerted similar effects as fresh NFL, indicating the storable NFL as a better substitute of Nanofat for clinical applications. This is the first report of NFL that provides substantial evidence of the anti-OA efficacy and mechanism and storable advantage of NFL, making it a promising remedy option for OA therapy in the clinic.

## Data Availability

The original contributions presented in the study are included in the article/Supplementary Material. The RNA-seq dataset presented in this study can be found in online repository, and the name of the repository and accession number can be found below: https://www.ncbi.nlm.nih.gov/geo; GSE200058. Further inquiries can be directed to the corresponding authors.

## Supplementary material

SUPPLEMENTARY FIGURE S1 General conditions and blood routine analysis of rats. **(A)** Rats body weight measurement; **(B)** Measurement of knee swelling in rats; **(C)** White blood cells count between NC, NF (Nanofat) and NFL group; **(D)** Monocytes count between NC, NF (Nanofat) and NFL group; **(E)** Lymphocytes count between NC, NF (Nanofat) and NFL group; **(F)** Neutrophils count between NC, NF (Nanofat) and NFL group. **p* < 0.05 *vs*. NC group.

SUPPLEMENTARY FIGURE S2 The cell viability of chondrocytes after NFL, exo (exosome) and TP (total protein) treatment. **(A)** The proliferative rate of chondrocytes at 24 h; **(B)** The proliferative rate of chondrocytes at 48 h. Values were shown as mean ± SD. **p* < 0.05 *vs*. TNF-α group; ***p* < 0.01 *vs*. TNF-α group; ****p* < 0.001 *vs*. TNF-α group.

SUPPLEMENTARY FIGURE S3 Western blot analysis of chondrocytes in TGF-β-Smad2/3 signaling pathway. **(A)** Western blot profile of protein bands; **(B)** statistical analysis of Western blot protein bands; **(C)** Western blot profile of protein bands; **(D)** Expressions of phospho-Smad2 in rat chondrocytes. **(E)** Expressions of phospho-Smad3 in rat chondrocytes. **(F)** Expressions of Smad2/3 in rat chondrocytes. ^##^ *p* < 0.01 *vs*. NC group; **p* < 0.05, ***p* < 0.01 or ****p* < 0.001 *vs*. TNF-α group.

SUPPLEMENTARY FIGURE S4
**(A)** Sketch map of NFL production.

SUPPLEMENTARY TABLE S1 The details of DEGs in RNA-seq.
