# Corrigendum: Nanofat lysate ameliorates pain and cartilage degradation of osteoarthritis through activation of TGF-β-Smad2/3 signaling of chondrocytes

**DOI:** 10.3389/fphar.2024.1401506

**Published:** 2024-06-13

**Authors:** Yanzhi Ge, Wenting Xu, Zuxiang Chen, Haiyan Zhang, Wenbo Zhang, Junjie Chen, Jiefeng Huang, Wenxi Du, Peijian Tong, Letian Shan, Li Zhou

**Affiliations:** ^1^ The First Affiliated Hospital, Zhejiang Chinese Medical University, Hangzhou, Zhejiang, China; ^2^ Zhejiang Hospital, Hangzhou, Zhejiang, China; ^3^ Cell Resource Bank and Integrated Cell Preparation Center of Xiaoshan District, Hangzhou Regional Cell Preparation Center (Sanjiang Shangyu Biotechnology Co., Ltd.), Hangzhou, China; ^4^ Department of Rheumatism Immunology, Changhai Hospital, Navy Medical University, Shanghai, China

**Keywords:** osteoarthritis, Nanofat lysate, TGF-β–Smad2/3 signaling, chondrocytes, RNA sequencing

In the published article, there was an error in Figure 3 as published. In the left panel of Figure 3B photo for the TNF-α+NF group was erroneously used as the photo for the TNF-α+NFL group. The corrected Figure 3 and its caption appear below.

**FIGURE 3:**
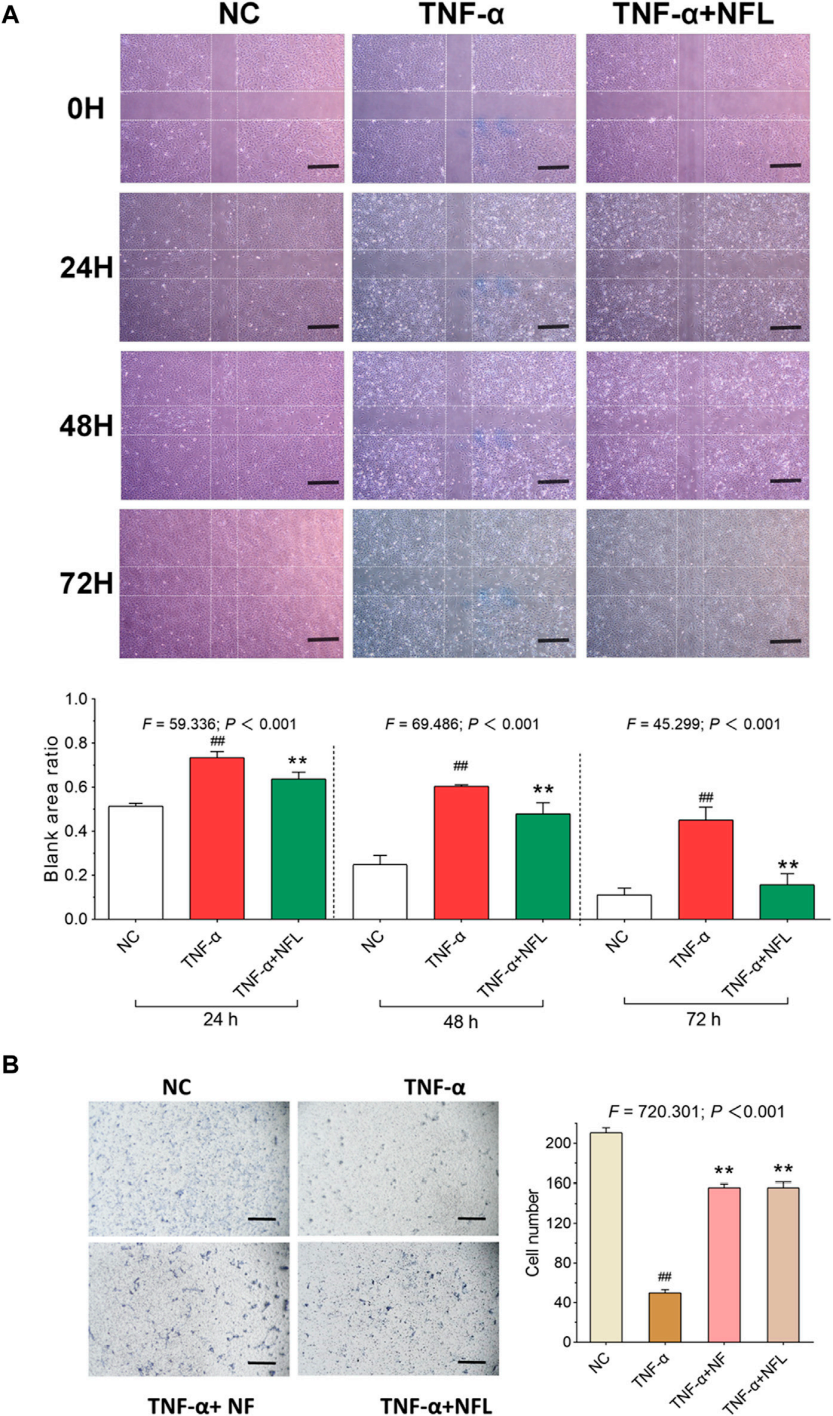
Wound healing and cell migration assays on chondrocytes with NFL treatment. **(A)** Wounding healing assay of chondrocytes with NFL treatment at 24, 48, and 72 h, and wound healing rate represented as the ratio of the scratched wound area at 24, 48, and 72 h treatment to the area without treatment (0 h). **(B)** Transwell assay of chondrocytes with NF and NFL intervention. Values are shown as mean ± SD. ##*p* < 0.01 vs. NC group; **p < 0.01 vs. TNF-α group. NF, Nanofat. Scale bar = 200 μm.

In the published article, there was an error. An old laboratory animal production license number was erroneously included in place of the new one.

A correction has been made to **2 Materials and Methods**, *2.2 Animal preparation*, Paragraph Number 1. This sentence previously stated:

“A total of 50 male Sprague-Dawley (SD) rats weighing 180–220 g were purchased from Shanghai Super B&K Laboratory Animal Co. Ltd. [Grade SPF II, SCXK (Shanghai): 2013-0016].”

The corrected sentence appears below:

“A total of 50 male Sprague-Dawley (SD) rats weighing 180–220 g were purchased from Shanghai Super B&K Laboratory Animal Co. Ltd. [Grade SPF II, SCXK (Shanghai): 2018-0006].”

In the published article, there was an error. The project number of the animal study was incorrectly used as the animal ethics approval number.

A correction has been made to **2 Materials and Methods**, *2.2 Animal preparation*, Line 9 to 13 in the paragraph. This sentence previously stated:

“All the experimental procedures were strictly in accordance with the Chinese legislation on the use and care of laboratory animals and approved by the Medical Norms and Ethics Committee of Zhejiang Chinese Medical University (Approval number: 10890).”

The corrected sentence appears below:

“All the experimental procedures were strictly in accordance with the Chinese legislation on the use and care of laboratory animals and approved by the Medical Norms and Ethics Committee of Zhejiang Chinese Medical University (Approval number: IACUC-20190506-14).”

The authors apologize for these errors and state that this does not change the scientific conclusions of the article in any way. The original article has been updated.

