# Supplementary figures and images for: Nanofat lysate ameliorates pain and cartilage degradation of osteoarthritis through activation of TGF-β–Smad2/3 signaling of chondrocytes
Source: Front Pharmacol. 2023 Mar 27;14:900205. doi: 10.3389/fphar.2023.900205 (PMC10083246; doi:10.3389/fphar.2023.900205)

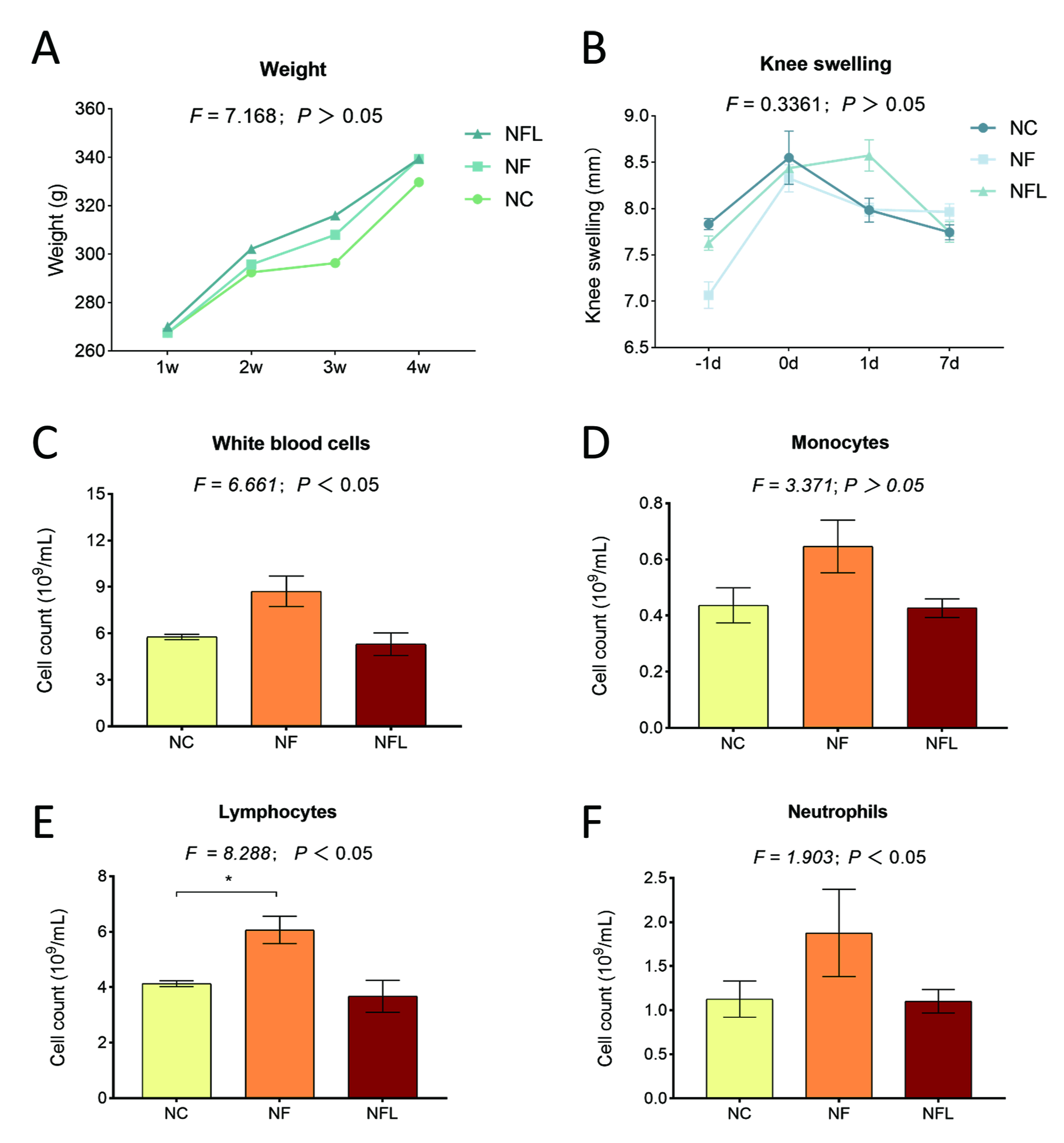

Supplement: Supplementary file 1 [file DataSheet1.zip › SUPPLEMENTARY DATA/FIGURE S1.TIF]

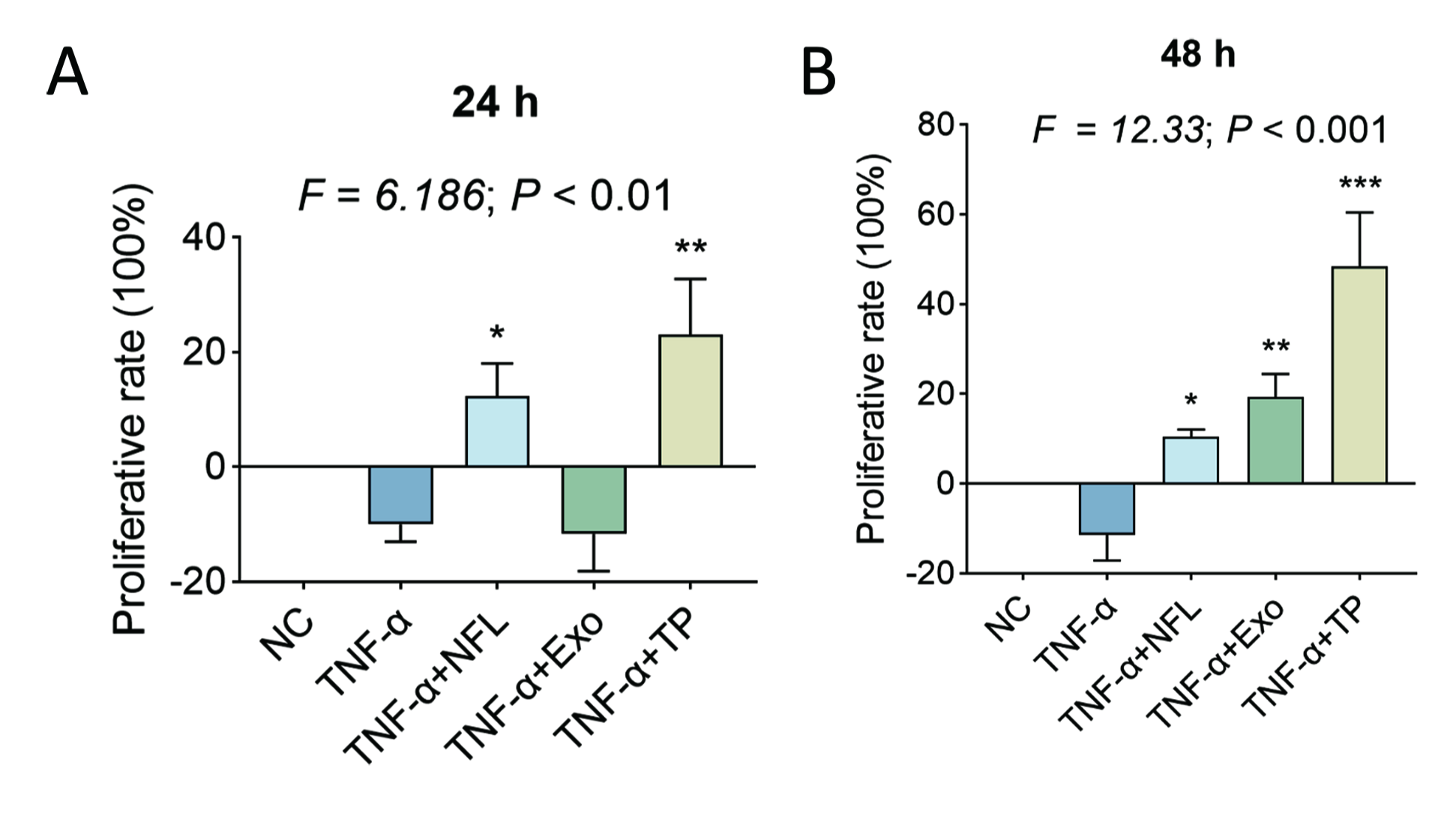

Supplement: Supplementary file 1 [file DataSheet1.zip › SUPPLEMENTARY DATA/FIGURE S2.TIF]

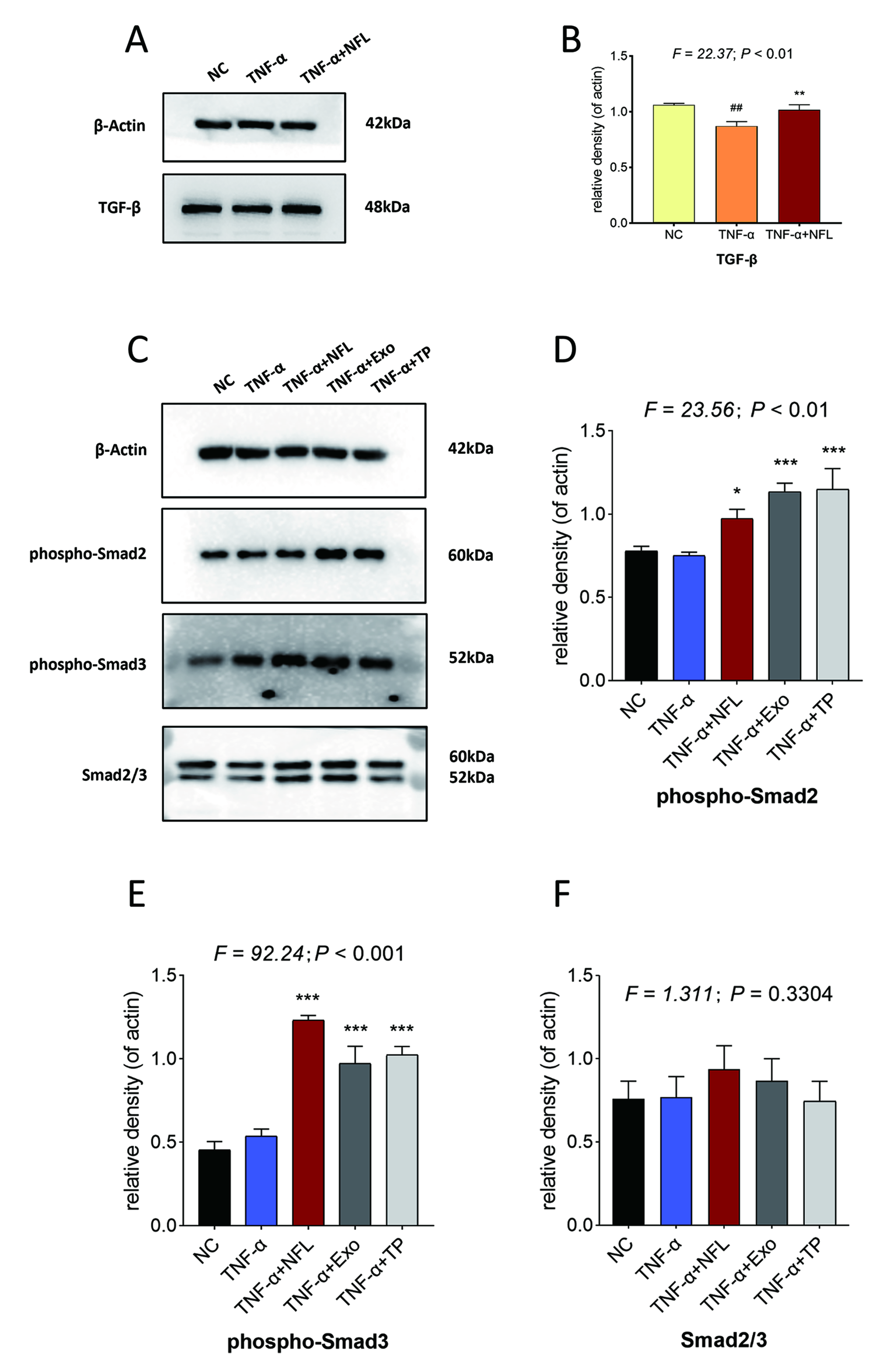

Supplement: Supplementary file 1 [file DataSheet1.zip › SUPPLEMENTARY DATA/FIGURE S3.TIF]

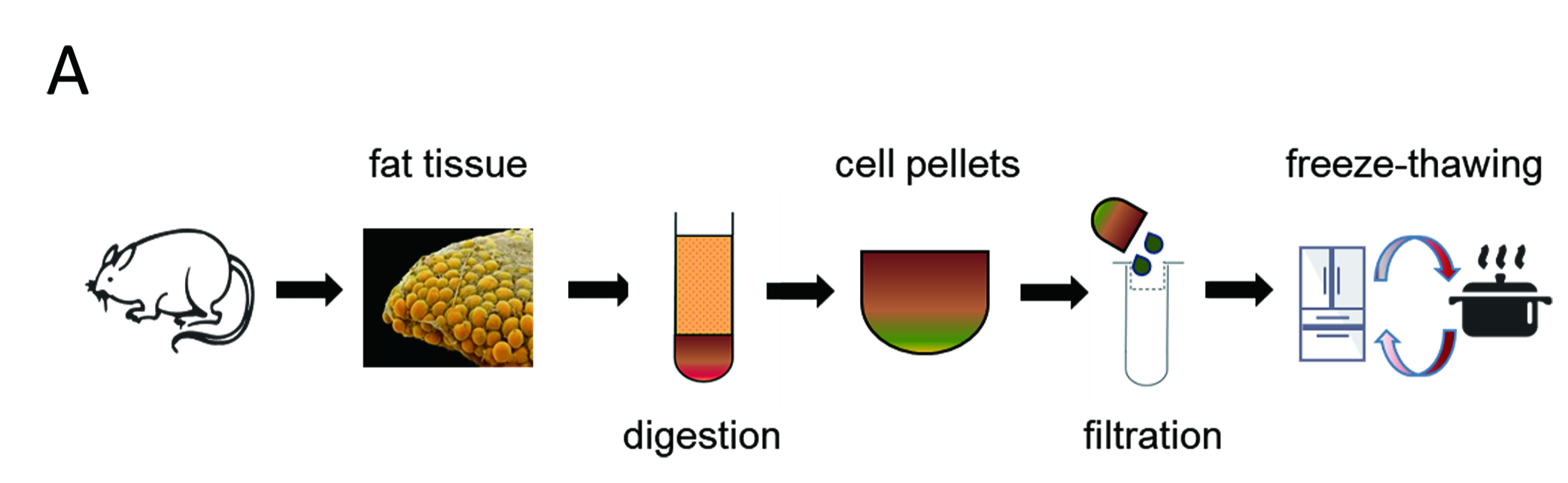

Supplement: Supplementary file 1 [file DataSheet1.zip › SUPPLEMENTARY DATA/FIGURE S4.TIF]
